# Action-Based Model Checking: Logic, Automata, and Reduction

**DOI:** 10.1007/978-3-030-53291-8_6

**Published:** 2020-06-16

**Authors:** Stephen F. Siegel, Yihao Yan

**Affiliations:** 8 Microsoft Research Lab, Redmond, WA USA; 9 University of Southern California, Los Angeles, CA USA; University of Delaware, Newark, DE 19716 USA

**Keywords:** Model checking, Action, Event, LTL, Stutter-invariant

## Abstract

Stutter invariant properties play a special role in state-based model checking: they are the properties that can be checked using partial order reduction (POR), an indispensable optimization. There are algorithms to decide whether an LTL formula or Büchi automaton (BA) specifies a stutter-invariant property, and to convert such a BA to a form that is appropriate for on-the-fly POR-based model checking.

The *interruptible* properties play the same role in action-based model checking that stutter-invariant properties play in the state-based case. These are the properties that are invariant under the insertion or deletion of “invisible” actions. We present algorithms to decide whether an LTL formula or BA specifies an interruptible property, and show how a BA can be transformed to an *interrupt normal form* that can be used in an on-the-fly POR algorithm. We have implemented these algorithms in a new model checker named McRERS, and demonstrate their effectiveness using the RERS 2019 benchmark suite.

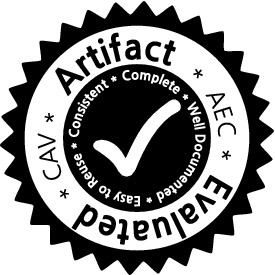



## Introduction

To apply model checking to a concurrent system, one must formulate properties that the system is expected to satisfy. A property may be expressed by specifying acceptable sequences of states, or by specifying acceptable sequences of actions—the events that cause the state to change. Each approach has advantages and disadvantages, and in any particular context one may be more appropriate than the other.

In the state-based context, there is a rich theory involving automata, logic, and reduction for model checking. Some of the core ideas in this theory can be summarized as follows. First, the behavior of the concurrent system is represented by a state-transition system *T*. One identifies a set $\textsf {AP}$ of atomic propositions, and each state of *T* is labeled by the set of propositions which hold at that state. An execution passes through an infinite sequence of states, which defines a *trace*, i.e., a sequence of subsets of $\textsf {AP}$. A *property* is a set of traces, and *T* satisfies the property if every trace of *T* is in *P*.

Properties may be specified by formulas in a temporal logic, such as LTL [26]. There are algorithms (e.g., [37]) to convert an LTL formula $\phi$ to an equivalent Büchi automaton (BA) $B_{\phi }$ with alphabet $2^{\textsf {AP}}$. (Properties may also be specified directly using BAs.) The system *T* satisfies $\phi$ if and only if the language of the synchronous product $T\otimes B_{\lnot \phi }$ is empty. The emptiness of the language can be determined on-the-fly, i.e., while the reachable states of the product are being constructed.

A property *P* is *stutter-invariant* if it is closed under the insertion and deletion of repetitions, i.e., $s_0s_1\cdots \in P \;\Leftrightarrow \;s_0^{i_0}s_1^{i_1}\cdots \in P$ holds for any positive integers $i_0,i_1,\cdots$. Many algorithms are known for deciding whether an LTL formula or a BA specifies a stutter-invariant property [22, 24]. There is also an argument that only stutter-invariant properties should be used in practice. For example, suppose that a trace is formed by sampling the state of a system once every millisecond. If we sample the same system twice each millisecond, and there are no state changes in the sub-millisecond intervals, the second trace will be stutter-equivalent to the first. A meaningful property should be invariant under this choice of time resolution.

Stutter-invariant properties are desirable for another reason: they admit the most significant optimization in model checking, partial order reduction (POR, [15, 23, 25]). At each state encountered in the exploration of the product space, an on-the-fly POR scheme produces a subset of the enabled transitions. Restricting the search to the transitions in those subsets does not affect the language emptiness question. Recent work has revealed that the BA must have a certain form—“SI normal form”—when POR is used with on-the-fly model checking, but any BA with a stutter-invariant language can be easily transformed into SI normal form [27].

The purpose of this paper is to elaborate an analogous theory for event-based models. Event-based models of concurrency are widely used and have been extremely influential for over three decades. For example, process algebras, such as CSP, are event-based and use *labeled transition systems* (LTSs) for the semantic model. Event-based models are the main formalism used in assume-guarantee reasoning (e.g, [10]), and in many other areas. There are mature model checking and verification tools for process algebras and LTSs, and which have significant industrial applications; see, e.g., [13]. Temporal logics, including LTL, CTL, and CTL*, have long been used to specify event-based systems [3, 7, 12].

We call the class of properties in the action context that are analogous to the stutter-invariant properties in the state context the *interruptible* properties (Sect. 3). These properties are invariant under “action stuttering” [34], i.e., the insertion or deletion of “invisible” actions. We present algorithms for deciding whether an LTL formula or a BA specifies an interruptible property (Theorems 1 and 2); to the best of our knowledge, these are the first published algorithms for deciding this property of formulas or automata.

Interruptible properties play the same role in action-based POR that stutter-invariant properties play in state-based POR. In particular, we present an action-based on-the-fly POR algorithm that works for interruptible properties (Sect. 4). As with the state-based case, the algorithm requires that the BA be in a certain normal form. We introduce a novel *interrupt normal form* (Definition 11) for this purpose, and show how any BA with an interruptible language can be transformed into that form. The relation to earlier work is discussed in Sect. 5. The effectiveness of these reduction techniques is demonstrated by applying them to problems in the 2019 RERS benchmark suite (Sect. 6).

## Preliminaries

Let *S* be a set. $2^{S}$ denotes the set of all subsets of *S*. $S^*$ denotes the set of finite sequences of elements of *S*; $S^\omega$ the infinite sequences. Let $\zeta =s_0s_1\cdots$ be a (finite or infinite) sequence and $i\ge 0$. If $\zeta$ is finite of length *n*, assume $i<n$. Then $\zeta (i)$ denotes the element $s_i$. For any $i\ge 0$, $\zeta ^i$ denotes the suffix $s_is_{i+1}\cdots$. ($\zeta ^i$ is empty if $\zeta$ is finite and $i\ge n$).

For $\zeta \in S^*$ and $\eta \in S^*\cup S^\omega$, $\zeta \circ \eta$ denotes the concatenation of $\zeta$ and $\eta$.

If $S\subseteq T$ and $\eta$ is a sequence of elements of *T*, $\eta |_S$ denotes the sequence obtained by deleting from $\eta$ all elements not in *S*.

### Linear Temporal Logic

Let ${\textsf {Act}}$ be a universal set of actions. We assume ${\textsf {Act}}$ is infinite.

#### Definition 1

${\mathsf {Form}}$ (the *LTL formulas over *${\textsf {Act}}$) is the smallest set satisfying:

- $${\mathsf {true}}\in {\mathsf {Form}}$$,
- if $a\in {\textsf {Act}}$ then $a\in {\mathsf {Form}}$, and
- if *f* and *g* are in ${\mathsf {Form}}$, so are $\lnot f$, $f\wedge g$, ${\mathbf {X}}{}f$, and $f{\mathbf {U}}{} g$.

Additional operators are defined as shorthand for other formulas: ${\mathsf {false}}= \lnot {\mathsf {true}}$, $f\vee g = \lnot ((\lnot f) \wedge \lnot g)$, $f\rightarrow g = (\lnot f)\vee g$, ${\mathbf {F}}{}f = {\mathsf {true}}{\mathbf {U}}{} f$, ${\mathbf {G}}{}f = \lnot {\mathbf {F}}\lnot f$, and $f{\mathbf {W}}g = (f{\mathbf {U}}g)\vee {\mathbf {G}}f$.    $\square$

#### Definition 2

The *alphabet* of an LTL formula *f*, denoted $\alpha f$, is the set of actions that occur syntactically within *f*.    $\square$

#### Definition 3

The *action-based semantics* of LTL is defined by the relation $\zeta ~\models _{\scriptscriptstyle {{\mathsf {A}}}~}f$, where $\zeta \in {\textsf {Act}}^\omega$ and $f\in {\mathsf {Form}}$, which is defined as follows:

- $$\zeta ~\models _{\scriptscriptstyle {{\mathsf {A}}}~}{\mathsf {true}}$$,
- $$\zeta ~\models _{\scriptscriptstyle {{\mathsf {A}}}~}a$$ iff $$\zeta (0)=a$$,
- $$\zeta ~\models _{\scriptscriptstyle {{\mathsf {A}}}~}\lnot f$$ iff $$\zeta \not \models _{\scriptscriptstyle {\mathsf {A}}}~f$$,
- $$\zeta ~\models _{\scriptscriptstyle {{\mathsf {A}}}~}f\wedge g$$ iff $$\zeta ~\models _{\scriptscriptstyle {{\mathsf {A}}}~}f$$ and $$\zeta ~\models _{\scriptscriptstyle {{\mathsf {A}}}~}g$$,
- $$\zeta ~\models _{\scriptscriptstyle {{\mathsf {A}}}~}{\mathbf {X}}f$$ iff $$\zeta ^1~\models _{\scriptscriptstyle {{\mathsf {A}}}~}f$$, and
- $$\zeta ~\models _{\scriptscriptstyle {{\mathsf {A}}}~}f{\mathbf {U}}{} g$$ iff $$\exists i\ge 0\, .\, (\zeta ^i~\models _{\scriptscriptstyle {{\mathsf {A}}}~}g\, \wedge \, \forall j\in 0..i-1\, .\, \zeta ^j~\models _{\scriptscriptstyle {{\mathsf {A}}}~}f)$$.    $$\square $$

When using the action-based semantics, the logic is sometimes referred to as “Action LTL” or ALTL [11, 12].

The *state-based semantics* is defined by a relation $\xi ~\models _{\scriptscriptstyle {{\mathsf {S}}}~}f$, where $\xi \in (2^{{\textsf {Act}}})^\omega$. The definition of $~\models _{\scriptscriptstyle {{\mathsf {S}}}~}$ is well-known, and is exactly the same as Definition 3, except that $\xi ~\models _{\scriptscriptstyle {{\mathsf {S}}}~}a$ iff $a\in \xi (0)$. The action semantics are consistent with the state semantics in the following sense. Let $f\in {\mathsf {Form}}$, and $\zeta =a_0a_1\cdots \in {\textsf {Act}}^{\omega }$. Let $\xi =\{a_0\}\{a_1\}\cdots \in (2^{{\textsf {Act}}})^\omega$. Then $\zeta ~\models _{\scriptscriptstyle {{\mathsf {A}}}~}f$ iff $\xi ~\models _{\scriptscriptstyle {{\mathsf {S}}}~}f$. The main difference between the state- and action-based formalisms is that in the state-based formalism, any number of atomic propositions can hold at each step. In the action-based formalism, precisely one action occurs in each step.

#### Definition 4

Let $$f,g\in {\mathsf {Form}}$$. Define

- (action equivalence) $f\equiv _{\scriptscriptstyle {{\mathsf {A}}}}g$ if $(\zeta ~\models _{\scriptscriptstyle {{\mathsf {A}}}~}f \;\Leftrightarrow \;\zeta ~\models _{\scriptscriptstyle {{\mathsf {A}}}~}g)$ for all $\zeta \in {\textsf {Act}}^\omega$
- (state equivalence) $f\equiv _{\scriptscriptstyle {{\mathsf {S}}}}g$ if $(\xi ~\models _{\scriptscriptstyle {{\mathsf {S}}}~}f \;\Leftrightarrow \;\xi ~\models _{\scriptscriptstyle {{\mathsf {S}}}~}g)$ for all $\xi \in (2^{{\textsf {Act}}})^\omega$.    $\square$

The following fact about the state-based semantics can be proved by induction on the formula structure:

#### Lemma 1

Let $f\in \mathsf {Form}$ and $\xi =s_0s_1\cdots \in (2^{\mathsf {Act}})^\omega$. Let $\xi '=s'_0s'_1\cdots$, where $s'_i=\alpha f \cap s_i$. Then $\xi ~\models _{\scriptscriptstyle {{\mathsf {S}}}~}f$ iff $\xi '~\models _{\scriptscriptstyle {{\mathsf {S}}}~}f$.

The following shows that action LTL, like ordinary state-based LTL, is a decidable logic:

#### Proposition 1

Let $$f,g\in {\mathsf {Form}}$$, $$A=\alpha f \cup \alpha g$$, and

$$ h = {\mathbf {G}}\Big [ \big ( \bigwedge _{a\in A}\lnot a \big ) \vee \bigvee _{a\in A} \big ( a\wedge \bigwedge _{b\in A\setminus \{a\}}\lnot b \big ) \Big ]. $$

Then $f\equiv _{\scriptscriptstyle {{\mathsf {A}}}}g \;\Leftrightarrow \;f\wedge h \equiv _{\scriptscriptstyle {{\mathsf {S}}}}g\wedge h$. In particular, action equivalence is decidable.

#### Proof

Note the meaning of *h*: at each step in a state-based trace, at most one element of *A* is true.

Suppose $f\wedge h \equiv _{\scriptscriptstyle {{\mathsf {S}}}}g\wedge h$. Let $\zeta =a_0a_1\cdots \in {\textsf {Act}}^\omega$. Let $\xi =\{a_0\}\{a_1\}\cdots$. We have $\xi ~\models _{\scriptscriptstyle {{\mathsf {S}}}~}h$. By the consistency of the state and action semantics, we have

$$ \zeta ~\models _{\scriptscriptstyle {{\mathsf {A}}}~}f \;\Leftrightarrow \;\xi ~\models _{\scriptscriptstyle {{\mathsf {S}}}~}f \;\Leftrightarrow \;\xi ~\models _{\scriptscriptstyle {{\mathsf {S}}}~}f\wedge h \;\Leftrightarrow \;\xi ~\models _{\scriptscriptstyle {{\mathsf {S}}}~}g\wedge h \;\Leftrightarrow \;\xi ~\models _{\scriptscriptstyle {{\mathsf {S}}}~}g \;\Leftrightarrow \;\zeta ~\models _{\scriptscriptstyle {{\mathsf {A}}}~}g, $$

hence $$f\equiv _{\scriptscriptstyle {{\mathsf {A}}}}g$$.

Suppose instead that $f\equiv _{\scriptscriptstyle {{\mathsf {A}}}}g$. We wish to show $\xi ~\models _{\scriptscriptstyle {{\mathsf {S}}}~}f\wedge h \;\Leftrightarrow \;\xi ~\models _{\scriptscriptstyle {{\mathsf {S}}}~}g\wedge h$ for any $\xi =s_0s_1\cdots \in (2^{{\textsf {Act}}})^\omega$. By Lemma 1, it suffices to assume $s_i\subseteq A$ for all *i*.

Let $\tau$ be any element of ${\textsf {Act}}\setminus A$. (Here we are using the fact that ${\textsf {Act}}$ is infinite, while *A* is finite.) If $|s_i|>1$ for some *i*, then $\xi$ violates *h* and therefore violates both $f\wedge h$ and $g\wedge h$. So suppose $|s_i|\le 1$ for all *i*, which means $\xi ~\models _{\scriptscriptstyle {{\mathsf {S}}}~}h$. Let $\zeta =a_0a_1\cdots$, where $a_i$ is the sole member of $s_i$ if $|s_i|=1$, or $\tau$ if $|s_i|=0$. By Lemma 1, $\xi ~\models _{\scriptscriptstyle {{\mathsf {S}}}~}f$ iff $\{a_0\}\{a_1\}\cdots \models _{\scriptscriptstyle {{\mathsf {S}}}~}f$. By the consistency of the action and state semantics, this is equivalent to $\zeta ~\models _{\scriptscriptstyle {{\mathsf {A}}}~}f$. A similar statement holds for *g*. Hence

$$ \xi ~\models _{\scriptscriptstyle {{\mathsf {S}}}~}f\wedge h \;\Leftrightarrow \;\xi ~\models _{\scriptscriptstyle {{\mathsf {S}}}~}f \;\Leftrightarrow \;\zeta ~\models _{\scriptscriptstyle {{\mathsf {A}}}~}f \;\Leftrightarrow \;\zeta ~\models _{\scriptscriptstyle {{\mathsf {A}}}~}g \;\Leftrightarrow \;\xi ~\models _{\scriptscriptstyle {{\mathsf {S}}}~}g \;\Leftrightarrow \;\xi ~\models _{\scriptscriptstyle {{\mathsf {S}}}~}g\wedge h. $$

The proposition reduces the question of action equivalence to one of ordinary (state) equivalence of LTL formulas, which is known to be decidable ( [26], see also [36, Thm. 24]).    $\square$

#### Definition 5

For $A\subseteq {\textsf {Act}}$ and $f\in {\mathsf {Form}}$ with $\alpha f\subseteq A$, let

$$\mathcal {L}(f,A) = \{\zeta \in A^\omega \mid \zeta ~\models ~f\}.$$

   $$\square $$

### Büchi Automata

#### Definition 6

A *Büchi Automaton* (BA) over ${\textsf {Act}}$ is a tuple $(S,\varSigma ,\rightarrow ,S^{0},F)$ where

1. *S* is a finite set of *states*,
2. $\varSigma$, the *alphabet*, is a finite subset of ${\textsf {Act}}$,
3. $\rightarrow \subseteq S\times \varSigma \times S$ is the *transition relation*,
4. $S^{0}\subseteq S$ is the set of *initial states*, and
5. $F\subseteq S$ is the set of *accepting states*.    $\square$

We will use the following notation and terminology for a BA *B*. The *source* of a transition $(s,a,s')$ is *s*, the *destination* is $s'$, and the *label* is *a*. We write $s\xrightarrow {a}s'$ as shorthand for $(s,a,s')\in \rightarrow$, and $s\xrightarrow {a_{0}a_{1}\ldots a_{n}}s'$ for $\exists s_{1},s_{2},\ldots s_{n}\in S\, .\, s\xrightarrow {a_{0}}s_{1}\xrightarrow {a_{1}}s_{2}\ldots s_{n}\xrightarrow {a_{n}}s'$. For $a\in A$ and $s\in S$, we say *a* is *enabled at s* if $s{\mathop {\rightarrow }\limits ^{a}}s'$ for some $s'\in S$. The set of all actions enabled at *s* is denoted $\textsf {enabled}(B,s)$.

For $s\in S$, a *path in B* *starting from s* is a (finite or infinite) sequence $\pi$ of transitions such that (1) if $\pi$ is not empty, the source of $\pi (0)$ is *s*, and (2) the destination of $\pi (i)$ is the source of $\pi (i+1)$ for all *i* for which these are defined. If $\pi$ is not empty, define ${\mathsf {first}}(\pi )$ to be *s*; if $\pi$ is finite, define ${\mathsf {last}}(\pi )$ to be the destination of the last transition of $\pi$. We say $\pi$ *spells the word* $a_0a_1\cdots$, where $a_i$ is the label of $\pi (i)$.

An infinite path is *accepting* if it visits a state in *F* infinitely often. An *(accepting) trace starting from s* is a word spelled by an (accepting) path starting from *s*. An *(accepting) trace of B* is an (accepting) trace starting from an initial state. The *language of B*, denoted $\mathcal {L}(B)$, is the set of all accepting traces of *B*.

#### Proposition 2

There is an algorithm that consumes any finite subset *A* of $\mathsf {Act}$ and an $f\in {\mathsf {Form}}$ with $\alpha f\subseteq A$, and produces a BA *B* with alphabet *A* such that $\mathcal {L}(B)=\mathcal {L}(f,A)$.

#### Proof

There are well-known algorithms to produce a BA *C* with alphabet $2^{A}$ which accepts exactly the words satisfying *f* under the state semantics (e.g., [37]). Let *B* be the same as *C*, except the alphabet is *A* and there is a transition $s\xrightarrow {a}s'$ in *B* iff there is a transition $s\xrightarrow {\{a\}}s'$ in *C*. We have

$$\begin{aligned} a_0a_1\cdots \in \mathcal {L}(B)&\;\Leftrightarrow \;\{a_0\}\{a_1\}\cdots \in \mathcal {L}(C)\\&\;\Leftrightarrow \;\{a_0\}\{a_1\}\cdots ~\models _{\scriptscriptstyle {{\mathsf {S}}}~}f\\&\;\Leftrightarrow \;a_0a_1\cdots \in \mathcal {L}(f,A). \end{aligned}$$

   $$\square $$

In practice, tools that convert LTL formulas to BAs produce an automaton in which an edge is labeled by a propositional formula $\phi$ over $\alpha f$. Such an edge represents a set of transitions, one for each $P\subseteq A$ for which $\phi$ holds for the valuation that assigns $\textit{true}$ to each element of *P* and $\textit{false}$ to each element of $A\setminus P$. In this case, the conversion to *B* entails creating one transition for each $a\in A$ for which $\phi$ holds when $\textit{true}$ is assigned to *a* and $\textit{false}$ is assigned to all other actions.

#### Definition 7

Let $B_{i} = (S_i,\varSigma _i,\rightarrow _i, S^{0}_i,F_i)$ ($i=1,2$) denote two BAs over ${\textsf {Act}}$. The *parallel composition of *$B_1$ *and* $B_2$ is the BA

$$ B_1\parallel B_2\equiv (S_1\times S_2,\varSigma _1\cup \varSigma _2,\rightarrow , S^{0}_1\times S^{0}_2,F_1\times F_2), $$

where $\rightarrow$ is defined by

$$ \displaystyle \frac{s_1\xrightarrow {a}_1s_1' \,\,\, a\not \in \varSigma _2}{\langle s_1,s_2\rangle \xrightarrow {a} \langle s_1',s_2\rangle } \,\,\,\,\,\,\, \frac{s_2\xrightarrow {a}_2s_2' \,\,\, a\not \in \varSigma _1}{\langle s_1,s_2\rangle \xrightarrow {a} \langle s_1,s_2'\rangle } \,\,\,\,\,\,\, \frac{s_1\xrightarrow {a}_1s_1' \,\,\, s_2\xrightarrow {a}_2s_2'}{\langle s_1,s_2\rangle \xrightarrow {a} \langle s_1',s_2'\rangle } .$$

   $$\square $$

If we flatten all tuples (e.g., identify $(S_1\times S_2)\times S_3$ with $S_1\times S_2\times S_3$) then $\parallel$ is an associative operator.

Note that in the special case where the two automata have the same alphabet ($\varSigma _1=\varSigma _2$), every action is synchronizing, and the parallel composition is the usual “synchronous product.” In this case, $\mathcal {L}(B_1\parallel B_2)=\mathcal {L}(B_1)\cap \mathcal {L}(B_2)$.

### Labeled Transition Systems

#### Definition 8

A *labeled transition system* (LTS) over ${\textsf {Act}}$ is a tuple $(Q,A,\rightarrow ,q^{0})$ for which $(Q,A,\rightarrow ,\{q^0\},Q)$ is a BA over ${\textsf {Act}}$. In other words, it is a BA in which all states are accepting and there is only one initial state.    $\square$

#### Definition 9

Let *M* be an LTS with alphabet *A*, and *f* an LTL formula with $\alpha f\subseteq A$. We write $M~\models ~f$ if $\mathcal {L}(M)\subseteq \mathcal {L}(f,A)$.    $\square$

The following observation is the basis of the automata-theoretic approach to model checking (cf. [36, §4.2]):

#### Proposition 3

Let *M* be an LTS with alphabet *A* and *f* an LTL formula with $\alpha f\subseteq A$. Let *B* be a BA with $\mathcal {L}(B)=\mathcal {L}(\lnot f,A)$. Then $M~\models ~f\;\Leftrightarrow \;\mathcal {L}(M\parallel B)=\emptyset$.

#### Proof

*M* and *B* have the same alphabet, so $\mathcal {L}(M\parallel B)=\mathcal {L}(M)\cap \mathcal {L}(B)$, hence

$$ \mathcal {L}(M\parallel B) =\mathcal {L}(M)\cap \mathcal {L}(\lnot f, A) =\mathcal {L}(M)\cap (A^\omega \setminus \mathcal {L}(f,A)) =\mathcal {L}(M)\setminus \mathcal {L}(f,A). $$

This set is empty iff $\mathcal {L}(M)\subseteq \mathcal {L}(f,A)$.    $\square$

There are various algorithms to determine language emptiness of a BA; in this paper we use the well-known Nested Depth First Search (NDFS) algorithm [2].

## Interruptible Properties

### Definition and Examples

An LTS comes with an alphabet, which is a subset *A* of ${\textsf {Act}}$. By a *property over A* we simply mean a subset *P* of $A^\omega$. We say a trace $\zeta \in A^\omega$ *satisfies* *P* if $\zeta \in P$. We have already seen two ways to specify properties. An LTL formula *f* with $\alpha f\subseteq A$ specifies the property $\mathcal {L}(f,A)$. A Büchi automaton *B* with alphabet *A* specifies the property $\mathcal {L}(B)$. We next define a special class of properties:

#### Definition 10

Given sets $V\subseteq A\subseteq {\textsf {Act}}$, we say a property *P* over *A* is *V-interruptible* if

$$ \zeta |_V=\eta |_V \Rightarrow (\zeta \in P \;\Leftrightarrow \;\eta \in P) \qquad \quad \text {for all }\zeta ,\eta \in A^\omega . $$

An LTL formula *f* is *V-interruptible* if $\mathcal {L}(f,{\textsf {Act}})$ is *V*-interruptible. We say *f* is *interruptible* if *f* is $\alpha f$-interruptible. The set of all interruptible LTL formulas is denoted ${\mathsf {Intrpt}}$.    $\square$

The set *V* is known as the *visible set*. The definition essentially says that the insertion or deletion of invisible actions (those in $A\setminus V$) has no bearing on whether a trace satisfies *P*. Put another way, the question of whether a trace belongs to *P* is determined purely by its visible actions. The following collects some basic facts about interruptibility. All follow immediately from the definitions.

#### Proposition 4

Let $V\subseteq A\subseteq \mathsf {Act}$, $P\subseteq A^\omega$ and $f,g\in {\mathsf {Form}}$. Then all of the following hold:

1. *P* is *A*-interruptible.
2. If *P* is *V*-interruptible, and $V\subseteq V'$, then *P* is $V'$-interruptible.
3. If *f* is interruptible and $\alpha f\subseteq A$, then $\mathcal {L}(f,A)$ is $\alpha f$-interruptible.
4. *f* is interruptible iff the following holds:
  $$ \forall \zeta ,\eta \in \mathsf {Act}^\omega \,.\, ( \zeta |_{\alpha f}=\eta |_{\alpha f} \wedge \zeta ~\models _{\scriptscriptstyle {{\mathsf {A}}}~}f ) \Rightarrow \eta ~\models _{\scriptscriptstyle {{\mathsf {A}}}~}f. $$
5. If $\alpha f=\alpha g$ and $f\equiv _{\scriptscriptstyle {{\mathsf {A}}}}g$ then *f* is interruptible iff *g* is interruptible.

Many, if not most, properties that arise in practice are *V*-interruptible for the set *V* of actions that are mentioned in the property. Assuming *a*, *b*, and *c* are distinct actions, we have:

- For any $n\ge 0$, the property “*a* occurs at most *n* times” is $\{a\}$-interruptible, since the insertion or deletion of actions other than *a* cannot affect whether a word satisfies that property. The same is true for the properties “*a* occurs at least *n* times” and “*a* occurs exactly *n* times.” These are examples of the *bounded existence pattern with global scope* in a widely used property specification pattern system [5]. LTL formulas in this category include ${\mathbf {G}}\lnot a$ (*a* occurs 0 times), ${\mathbf {F}}{}a$ (*a* occurs at least once), and ${\mathbf {F}}(a\wedge {\mathbf {X}}{\mathbf {F}}{}a)$ (*a* occurs at least twice).
- The property “after any occurrence of *a*, *b* eventually occurs”, ${\mathbf {G}}(a\rightarrow {\mathbf {F}}b)$, is $\{a,b\}$-interruptible. This is the *response pattern with global scope* [5].
- The property “after any occurrence of *a*, *c* will eventually occur, and no *b* will occur until *c*”, ${\mathbf {G}}(a\rightarrow ((\lnot b){\mathbf {U}}{}c))$, is $\{a,b,c\}$-interruptible. This is a variation on the *absence pattern with after-until scope*, and is used to specify mutual exclusion [5].

On the other hand, the property “*a* occurs at time 0”, (LTL formula *a*) is not $\{a\}$-interruptible. Neither is “an event other than *a* occurs at least once” (${\mathbf {F}}\lnot a$) nor “only *a* occurs” (${\mathbf {G}}{}a$). The property “every occurrence of *a* is followed immediately by *b*,” formula ${\mathbf {G}}(a\rightarrow {\mathbf {X}}{}b)$, is not $\{a,b\}$-interruptible. The property “after any occurrence of *a*, *c* eventually occurs and until then only *b* occurs,” ${\mathbf {G}}(a\rightarrow {\mathbf {X}}(b{\mathbf {U}}{}c))$, is not $\{a,b,c\}$-interruptible.

The following provides a useful way to show that two interruptible properties are equal:

#### Lemma 2

Suppose $V\subseteq A\subseteq \mathsf {Act}$ and $P_1$ and $P_2$ are *V*-interruptible properties over *A*. Let $\mathcal {F} = V^{\omega } \, \cup \, V^*\circ (A\setminus V)^{\omega }.$ Then $P_1=P_2$ iff $P_1\cap \mathcal {F}=P_2\cap \mathcal {F}$.

#### Proof

Assume $P_1\cap \mathcal {F}=P_2\cap \mathcal {F}$. Let $\zeta \in P_1$. If $\zeta |_V$ is infinite, then since $\zeta |_V|_V=\zeta |_V$, and $P_1$ is *V*-interruptible, $\zeta |_V\in P_1$. But $\zeta |_V\in V^\omega$, so $\zeta |_V\in P_1\cap \mathcal {F}$, and therefore $\zeta |_V\in P_2$. Since $P_2$ is *V*-interruptible, $\zeta \in P_2$.

If $\zeta |_V$ is finite, there is a prefix $\theta$ of $\zeta$ such that $\zeta =\theta \circ \eta$, with $\eta \in (V\setminus A)^\omega$. Let $\xi =\theta |_V\circ \eta$. We have $\xi \in V^*\circ (A\setminus V)^{\omega }$ and $\xi |_V = \zeta |_V$, hence $\xi \in P_1\cap \mathcal {F}$. Therefore $\xi \in P_2$, and since $P_2$ is *V*-interruptible, $\zeta \in P_2$.    $\square$

The elements of $\mathcal {F}$ are known as the *V*-*interrupt-free* words over *A*.

### Decidability of Interruptibility of LTL Formulas

We next show that interruptibility is a decidable property of LTL formulas. Define ${\textsf {intrpt}}:{\mathsf {Form}}\rightarrow {\mathsf {Form}}$ as follows. Given $f\in {\mathsf {Form}}$, let $V=\alpha f$ and $\hat{V}=\bigvee _{a\in V} a$, and define $\beta :{\mathsf {Form}}\rightarrow {\mathsf {Form}}$ by

$$\begin{aligned} \beta ({\mathsf {true}})&= {\mathsf {true}}\\ \beta (a)&= (\lnot \hat{V}) {\mathbf {U}}a \\ \beta (\lnot f_1)&= \lnot \beta (f_1) \\ \beta (f_1\wedge f_2)&= \beta (f_1)\wedge \beta (f_2)\\ \beta ({\mathbf {X}}f_1)&= ((\lnot \hat{V}){\mathbf {U}}(\hat{V}\wedge {\mathbf {X}}\beta (f_1))) \,\vee \, (({\mathbf {G}}\lnot \hat{V})\wedge {\mathbf {X}}\beta (f_1))\\ \beta (f_1{\mathbf {U}}f_2)&= \beta (f_1){\mathbf {U}}\beta (f_2). \end{aligned}$$

for $a\in {\textsf {Act}}$ and $f_1,f_2\in {\mathsf {Form}}$. Let ${\textsf {intrpt}}(f)=\beta (f)$.

#### Theorem 1

Let *f* be an LTL formula over $\mathsf {Act}$. The following hold:

1. $$\mathsf {intrpt}(f)$$ is interruptible.
2. *f* is interruptible iff $\mathsf {intrpt}(f)\equiv _{\scriptscriptstyle {{\mathsf {A}}}}f$.

In particular, interruptibility of LTL formulas is decidable.

Before proving Theorem 1, we give some intuition regarding the definition of ${\textsf {intrpt}}$. Function $\beta$ can be thought of as consuming a property on *V*-interrupt-free words (i.e., words in $V^\omega \cup V^*\circ (A\setminus V)^\omega$) and extending it to a property on all words ($A^\omega$). It is designed so that $\beta (g)$ is *V*-interruptible and agrees with *g* on *V*-interrupt-free words. For example, the formula *a* means “*a* is the first action” (in an interrupt-free word), which extends to the property “*a* is the first visible action” (in an arbitrary word). The formula ${\mathbf {X}}f_1$ states “$f_1$ holds after removing the first action,” so $\beta ({\mathbf {X}}f_1)$ should declare “$\beta (f_1)$ holds after removing the prefix ending in the first visible action.” That is almost correct, but there is also the possibility that an element of $A^\omega$ has no visible action, which is the reason for the second clause in the definition of $\beta ({\mathbf {X}}f_1)$.

The remainder of this subsection is devoted to the proof of Theorem 1. First note that ${\textsf {intrpt}}(f)$ and *f* have the same alphabet, i.e., $\alpha {\textsf {intrpt}}(f) = V$.

**Proof of Part 1.** Say a subformula *g* of *f* is *good* if $\beta (g)$ is *V*-interruptible, i.e.,

$$\begin{aligned} \forall \zeta ,\eta \in {\textsf {Act}}^\omega \,.\, \zeta |_V=\eta |_V \Rightarrow (\zeta ~\models _{\scriptscriptstyle {{\mathsf {A}}}~}\beta (g) \;\Leftrightarrow \;\eta ~\models _{\scriptscriptstyle {{\mathsf {A}}}~}\beta (g)). \end{aligned}$$

We show by induction on formula structure that every subformula of *f* is good. The case $g=f$ will show that ${\textsf {intrpt}}(f)$ is interruptible. Assume throughout that $\zeta |_V=\eta |_V$.

If $g={\mathsf {true}}$ then $\beta (g)={\mathsf {true}}$, so *g* is clearly good.

If $g=a$ for some $a\in {\textsf {Act}}$, then $\zeta ~\models _{\scriptscriptstyle {{\mathsf {A}}}~}\beta (g)=(\lnot \hat{V}){\mathbf {U}}a$ iff $\zeta |_V$ is non-empty and $\zeta |_V(0)=a$. Since this depends only on $\zeta |_V$, *g* is good.

If $g=\lnot f_1$ and $f_1$ is good, then *g* is good because

$$ \zeta ~\models _{\scriptscriptstyle {{\mathsf {A}}}~}\beta (g) \;\Leftrightarrow \;\zeta ~\not \models _{\scriptscriptstyle {\mathsf {A}}}~\beta (f_1) \;\Leftrightarrow \;\eta ~\not \models ~\beta (f_1) \;\Leftrightarrow \;\eta ~\models _{\scriptscriptstyle {{\mathsf {A}}}~}\beta (g). $$

If $g=f_1\wedge f_2$, and $f_1$ and $f_2$ are good, then *g* is good because

$$\begin{aligned} \zeta ~\models _{\scriptscriptstyle {{\mathsf {A}}}~}\beta (g)&\;\Leftrightarrow \;\zeta ~\models _{\scriptscriptstyle {{\mathsf {A}}}~}\beta (f_1) \,\wedge \, \zeta ~\models _{\scriptscriptstyle {{\mathsf {A}}}~}\beta (f_2)\\&\;\Leftrightarrow \;\eta ~\models _{\scriptscriptstyle {{\mathsf {A}}}~}\beta (f_1) \,\wedge \, \eta ~\models _{\scriptscriptstyle {{\mathsf {A}}}~}\beta (f_2) \;\Leftrightarrow \;\eta ~\models _{\scriptscriptstyle {{\mathsf {A}}}~}\beta (g). \end{aligned}$$

Suppose $g={\mathbf {X}}f_1$ and $f_1$ is good. There are two cases:

- **Case 1:** $\zeta |_V$ is empty. Then no suffix of $\zeta$ or $\eta$ satisfies $\hat{V}$. Hence
  $$ \theta ~\models _{\scriptscriptstyle {{\mathsf {A}}}~}\beta (g) \;\Leftrightarrow \;\theta ~\models _{\scriptscriptstyle {{\mathsf {A}}}~}{\mathbf {X}}\beta (f_1) \;\Leftrightarrow \;\theta ^1~\models _{\scriptscriptstyle {{\mathsf {A}}}~}\beta (f_1) \ \ \ \ \ (\theta \in \{\zeta ,\eta \}). $$
  Moreover, $\zeta ^1|_V=\eta ^1|_V$ (as both are empty), and $\beta (f_1)$ is good, so we have $\zeta ^1~\models _{\scriptscriptstyle {{\mathsf {A}}}~}\beta (f_1) \;\Leftrightarrow \;\eta ^1~\models _{\scriptscriptstyle {{\mathsf {A}}}~}\beta (f_1)$. These show $\zeta ~\models _{\scriptscriptstyle {{\mathsf {A}}}~}\beta (g) \;\Leftrightarrow \;\eta ~\models _{\scriptscriptstyle {{\mathsf {A}}}~}\beta (g)$.
- **Case 2:** $\zeta |_V$ is nonempty. Let *i* be the index of the first occurrence of an element of *V* in $\zeta$, and *j* the similar index for $\eta$. We have
  $$ \zeta ^{i+1}|_V = (\zeta |_V)^1 = (\eta |_V)^1 = \eta ^{j+1}|_V. $$
  As $f_1$ is good, it follows that $\zeta ^{i+1}~\models _{\scriptscriptstyle {{\mathsf {A}}}~}\beta (f_1) \;\Leftrightarrow \;\eta ^{j+1}~\models _{\scriptscriptstyle {{\mathsf {A}}}~}\beta (f_1).$ Hence
  $$ \zeta ~\models _{\scriptscriptstyle {{\mathsf {A}}}~}\beta (g) \;\Leftrightarrow \;\zeta ^{i+1}~\models _{\scriptscriptstyle {{\mathsf {A}}}~}\beta (f_1) \;\Leftrightarrow \;\eta ^{j+1}~\models _{\scriptscriptstyle {{\mathsf {A}}}~}\beta (f_1) \;\Leftrightarrow \;\eta ~\models _{\scriptscriptstyle {{\mathsf {A}}}~}\beta (g). $$

Suppose $g=f_1{\mathbf {U}}f_2$ and $f_1$ and $f_2$ are good. We have $\beta (g) = \beta (f_1){\mathbf {U}}\beta (f_2)$. If $\zeta ~\models _{\scriptscriptstyle {{\mathsf {A}}}~}\beta (g)$ then there exists $i\ge 0$ such that $\zeta ^i~\models _{\scriptscriptstyle {{\mathsf {A}}}~}\beta (f_2)$ and $\zeta ^j~\models _{\scriptscriptstyle {{\mathsf {A}}}~}\beta (f_1)$ for $j<i$. Now there is some $i'\ge 0$ such that $\eta ^{i'}|_V=\zeta ^i|_V$ and for all $j'<i'$, there is some $j<i$ such that $\eta ^{j'}|_V=\zeta ^j|_V$. It follows that $\eta \models \beta (g)$. Hence *g* is good.

**Proof of Part 2.** Suppose first that ${\textsf {intrpt}}(f)\equiv _{\scriptscriptstyle {{\mathsf {A}}}}f$. From part 1, ${\textsf {intrpt}}(f)$ is interruptible, so Proposition 4(5) implies *f* is interruptible.

Suppose instead that *f* is interruptible. We wish to show ${\textsf {intrpt}}(f)\equiv _{\scriptscriptstyle {{\mathsf {A}}}}f$. By Lemma 2, it suffices to show the two formulas agree on *V*-interrupt-free words. We will show by induction that for each subformula *g* of *f*, $\zeta ~\models _{\scriptscriptstyle {{\mathsf {A}}}~}g \;\Leftrightarrow \;\zeta ~\models _{\scriptscriptstyle {{\mathsf {A}}}~}\beta (g)$ for all *V*-interrupt-free $\zeta$. The case $g=f$ will complete the proof.

If $g={\mathsf {true}}$, $\beta (g)={\mathsf {true}}$ and the condition clearly holds.

If $g=a$ for some $a\in {\textsf {Act}}$, $\zeta ~\models _{\scriptscriptstyle {{\mathsf {A}}}~}\beta (g)\;\Leftrightarrow \;\zeta ~\models _{\scriptscriptstyle {{\mathsf {A}}}~}(\lnot \hat{V}){\mathbf {U}}a\;\Leftrightarrow \;\zeta ~\models _{\scriptscriptstyle {{\mathsf {A}}}~}a,$ as $\zeta$ is *V*-interrupt-free.

If $g=\lnot f_1$ and the inductive hypothesis holds for $f_1$, then

$$ \zeta ~\models _{\scriptscriptstyle {{\mathsf {A}}}~}\beta (g) \;\Leftrightarrow \;\zeta ~\not \models _{\scriptscriptstyle {\mathsf {A}}}~\beta (f_1) \;\Leftrightarrow \;\zeta ~\not \models _{\scriptscriptstyle {\mathsf {A}}}~f_1 \;\Leftrightarrow \;\zeta ~\models _{\scriptscriptstyle {{\mathsf {A}}}~}g. $$

If $g=f_1\wedge f_2$ and the inductive hypothesis holds for $f_1$ and $f_2$ then

$$ \zeta ~\models _{\scriptscriptstyle {{\mathsf {A}}}~}\beta (g) \;\Leftrightarrow \;\zeta ~\models _{\scriptscriptstyle {{\mathsf {A}}}~}\beta (f_1)\,\wedge \,\zeta ~\models _{\scriptscriptstyle {{\mathsf {A}}}~}\beta (f_2) \;\Leftrightarrow \;\zeta ~\models _{\scriptscriptstyle {{\mathsf {A}}}~}f_1 \,\wedge \, \zeta ~\models _{\scriptscriptstyle {{\mathsf {A}}}~}f_2 \;\Leftrightarrow \;\zeta ~\models _{\scriptscriptstyle {{\mathsf {A}}}~}g. $$

Suppose $g={\mathbf {X}}f_1$ and the inductive hypothesis holds for $f_1$. Note that any suffix of a *V*-interrupt-free word, e.g., $\zeta ^1$, is also *V*-interrupt-free. If $\zeta |_V$ is empty,

$$ \zeta ~\models _{\scriptscriptstyle {{\mathsf {A}}}~}\beta (g) \;\Leftrightarrow \;\zeta ~\models _{\scriptscriptstyle {{\mathsf {A}}}~}{\mathbf {X}}\beta (f_1) \;\Leftrightarrow \;\zeta ^1~\models _{\scriptscriptstyle {{\mathsf {A}}}~}\beta (f_1) \;\Leftrightarrow \;\zeta ^1~\models _{\scriptscriptstyle {{\mathsf {A}}}~}f_1 \;\Leftrightarrow \;\zeta ~\models _{\scriptscriptstyle {{\mathsf {A}}}~}g. $$

If $\zeta |_V$ is nonempty, then $\zeta ~\models _{\scriptscriptstyle {{\mathsf {A}}}~}\hat{V}$, so

$$\begin{aligned} \zeta ~\models _{\scriptscriptstyle {{\mathsf {A}}}~}\beta (g)&\;\Leftrightarrow \;\zeta ~\models _{\scriptscriptstyle {{\mathsf {A}}}~}(\lnot \hat{V}){\mathbf {U}}(\hat{V}\wedge {\mathbf {X}}\beta (f_1))\;\Leftrightarrow \;\zeta ~\models _{\scriptscriptstyle {{\mathsf {A}}}~}{\mathbf {X}}\beta (f_1)\\&\;\Leftrightarrow \;\zeta ^1~\models _{\scriptscriptstyle {{\mathsf {A}}}~}\beta (f_1)\;\Leftrightarrow \;\zeta ^1~\models _{\scriptscriptstyle {{\mathsf {A}}}~}f_1\;\Leftrightarrow \;\zeta ~\models _{\scriptscriptstyle {{\mathsf {A}}}~}g. \end{aligned}$$

If $g=f_1{\mathbf {U}}f_2$, then applying the inductive hypothesis to $f_1$ and $f_2$ yields

$$\begin{aligned} \zeta ~\models _{\scriptscriptstyle {{\mathsf {A}}}~}g&\;\Leftrightarrow \;\exists i>0 \,.\, \zeta ^i~\models _{\scriptscriptstyle {{\mathsf {A}}}~}f_2 \wedge \forall j<i \,.\, \zeta ^j~\models _{\scriptscriptstyle {{\mathsf {A}}}~}f_1\\&\;\Leftrightarrow \;\exists i>0 \,.\, \zeta ^i~\models _{\scriptscriptstyle {{\mathsf {A}}}~}\beta (f_2) \wedge \forall j<i \,.\, \zeta ^j~\models _{\scriptscriptstyle {{\mathsf {A}}}~}\beta (f_1)\\&\;\Leftrightarrow \;\zeta ~\models _{\scriptscriptstyle {{\mathsf {A}}}~}\beta (g). \end{aligned}$$

Decidability follows from part 2 and Proposition 1. This completes the proof of Theorem 1.

#### Remark 1

The definition of $\beta ({\mathbf {X}}f_1)$ is convenient for the proof but shorter definitions also work. If the formula $f_1$ is satisfied by some word $\zeta \in (A\setminus V)^{\omega }$, then all such $\zeta$ satisfy $f_1$, and the clause $({\mathbf {G}}\lnot \hat{V})\wedge {\mathbf {X}}\beta (f_1)$ can be replaced by ${\mathbf {G}}\lnot \hat{V}$. Otherwise, that clause can be removed altogether. One can determine whether a formula is satisfied by such a word by replacing every occurrence of every action with ${\mathsf {false}}$.

### Generation of Interruptible LTL Formulas

The following can be used to show that many formulas are interruptible. It establishes a kind of parity pattern involving a class of *positive* formulas (${\mathsf {Pos}}$) and a class of *negative* formulas (${\mathsf {Neg}}$). It is proved in [28].

#### Proposition 5

There exist ${\mathsf {Pos}},{\mathsf {Neg}}\subseteq {\mathsf {Form}}$ such that (i) for all $f,f'\in {\mathsf {Form}}$,

$$\begin{aligned} (f\in {\mathsf {Pos}}\wedge f'\equiv _{\scriptscriptstyle {{\mathsf {A}}}}f)&\Rightarrow f'\in {\mathsf {Pos}}\\ (f\in {\mathsf {Neg}}\wedge f'\equiv _{\scriptscriptstyle {{\mathsf {A}}}}f)&\Rightarrow f'\in {\mathsf {Neg}}, \end{aligned}$$

and (ii) for all $a\in \mathsf {Act}$, $f_1,f_2\in {\mathsf {Intrpt}}$, $g_1,g_2\in {\mathsf {Pos}}$, and $h_1,h_2\in {\mathsf {Neg}}$,

$$\begin{aligned} \textit{false},\; a,\; \lnot h_1,\; g_1\wedge g_2,\; g_1\vee g_2,\; a\wedge f_1,\; a\wedge {\mathbf {X}}f_1&\,\in \,{\mathsf {Pos}}\\ \textit{true},\; \lnot a,\; \lnot g_1,\; h_1\wedge h_2,\; h_1\vee h_2,\; \lnot a\vee f_1,\; \lnot a\vee {\mathbf {X}}f_1&\,\in \,{\mathsf {Neg}}\\ \textit{true},\; \textit{false},\; f_1\wedge f_2,\;f_1\vee f_2,\; \lnot f_1,\; {\mathbf {F}}g_1,\; {\mathbf {G}}h_1,\;f_1{\mathbf {U}}f_2,\; h_1{\mathbf {U}}g_1,\; h_1{\mathbf {U}}f_1&\,\in \,{\mathsf {Intrpt}}. \end{aligned}$$

Consider the examples from Sect. 3.1. The formula *a* is positive, so ${\mathbf {F}}{}a$ is interruptible. Since $\lnot a$ is negative, ${\mathbf {G}}\lnot a$ is interruptible. Since ${\mathbf {F}}{}a$ is interruptible, $a\wedge {\mathbf {X}}{\mathbf {F}}{}a$ is positive, hence ${\mathbf {F}}(a\wedge {\mathbf {X}}{\mathbf {F}}{}a)$ is interruptible.

Formula ${\mathbf {G}}(a\rightarrow {\mathbf {F}}b)$ is seen to be interruptible as follows. Since $b\in {\mathsf {Pos}}$, ${\mathbf {F}}b\in {\mathsf {Intrpt}}$, whence $\lnot a \vee {\mathbf {F}}b\in {\mathsf {Neg}}$. Since this last formula is action-equivalent to $a\rightarrow {\mathbf {F}}b$, we have $a\rightarrow {\mathbf {F}}b\in {\mathsf {Neg}}$. Therefore ${\mathbf {G}}(a\rightarrow {\mathbf {F}}b)\in {\mathsf {Intrpt}}$.

Similarly, $(\lnot b){\mathbf {U}}{}c\in {\mathsf {Intrpt}}$, so $a\rightarrow {\mathbf {X}}((\lnot b){\mathbf {U}}{}c)\in {\mathsf {Neg}}$. This negative formula is action-equivalent to $a\rightarrow ((\lnot b){\mathbf {U}}{}c)$, whence ${\mathbf {G}}(a\rightarrow ((\lnot b){\mathbf {U}}{}c))\in {\mathsf {Intrpt}}$.

Note that ${\mathsf {Intrpt}}$ and the set of stutter-invariant formulas are not comparable. For example, $f={\mathbf {F}}(a\wedge {\mathbf {X}}{\mathbf {F}}{}a)$ is interruptible, but not stutter-invariant. In fact *f* is not action-equivalent to any stutter-invariant formula *g*, since if there were such a *g*, the sequence $aab^\omega$ would satisfy *g*, but the stutter-equivalent sequence $ab^\omega$ cannot satisfy *g*. Conversely, the formulas *a* and ${\mathbf {G}}a$ are both stutter-invariant, but neither is interruptible. The formula ${\mathbf {F}}a$ is both stutter-invariant and interruptible. Finally, the formula ${\mathbf {X}}a$ is neither stutter-invariant nor interruptible.

### Decidability of Interruptibility of Büchi Automata

#### Definition 11

Let *B* be a BA with alphabet *A*, $V\subseteq A$ (the *visible* actions), and $I=A\setminus V$ (the *invisible actions*). We say *B* is in *V-interrupt normal form* if the following hold for any $x\in I$, $a\in A$, and states $s_1$, $s_2$, and $s_3$:

1. If $s_1{\mathop {\rightarrow }\limits ^{a}}s_2$ then *B* has a state $s_1'$ such that $s_1{\mathop {\rightarrow }\limits ^{x}}s_1'{\mathop {\rightarrow }\limits ^{a}}s_2$.
2. If $s_1{\mathop {\rightarrow }\limits ^{x}}s_2{\mathop {\rightarrow }\limits ^{a}}s_3$ then $s_1{\mathop {\rightarrow }\limits ^{a}}s_3$ and if $s_2$ is accepting then $s_1$ or $s_3$ is accepting.
3. If $s_1{\mathop {\rightarrow }\limits ^{x}}s_2$ then $s_1{\mathop {\rightarrow }\limits ^{y}}s_2$ for all $y\in I$.

#### Proposition 6

Suppose *B* is in *V*-interrupt normal form. Then $\mathcal {L}(B)$ is *V*-interruptible.

#### Proof

Suppose $\zeta ,\eta \in A^\omega$, $\zeta \in \mathcal {L}(B)$, and $\zeta |_V=\eta |_V$. We wish to show $\eta \in \mathcal {L}(B)$. Let $\pi$ be an accepting path for $\zeta$.

Assume $\zeta |_V$ is infinite. By Definition 11(2), we can remove all invisible transitions from the accepting path $\pi$, and the result is an accepting path that spells $\zeta |_V$. By Definition 11(1), we can insert any arbitrary finite sequence of invisible transition between two consecutive visible transitions; we can therefore construct an accepting path for $\eta$.

If $\zeta |_V$ is finite, proceed as above to form an accepting path which spells a finite prefix of $\eta$ followed by an infinite word of invisible actions. By Definition 11(3), that infinite suffix can be transformed to spell any infinite word of invisibles, and in that way one obtains an accepting path for $\eta$.    $\square$

Given any BA $B=(S,A,T,S^0,F)$ and a visible set $V\subseteq A$, define a BA ${\textsf {norm}}(B,V)$ as follows: if $V=A$, ${\textsf {norm}}(B,V)=B$, otherwise ${\textsf {norm}}(B,V)$ is $\hat{B}=(\hat{S},A,\hat{T},\hat{S}^0,\hat{F})$, where

$$\begin{aligned} D&= \{s\in S \mid \text {there is an accepting path from }s \text { with all labels in }I\} \\ \hat{S}&= \{\hat{u} \mid u\in S\} \cup \{u^{\sharp } \mid u\in F\setminus D\} \cup \{{\textsf {DIV}}\}\\ \hat{S}^0&= \{\hat{u}\mid u\in S^0\}\\ \hat{F}&= \{\hat{u}\mid u\in F\} \cup \{{\textsf {DIV}}\}\\ \hat{T}&= \begin{array}[t]{llllll} \{(\hat{u},a,\hat{v}) &{}\mid a\in V\wedge u,v\in S\wedge (u,a,v)\in T &{}\}\,\cup \\[0pt] \{(\hat{u},x,\hat{u}) &{}\mid x\in I \wedge u\in D\,\cup (S\setminus F) &{}\}\,\cup \\[0pt] \{({\textsf {DIV}},x,{\textsf {DIV}}) &{}\mid x\in I &{}\}\,\cup \\[0pt] \{(\hat{u},x,{\textsf {DIV}}) &{}\mid x\in I\wedge u\in D\setminus F &{}\}\,\cup \\[0pt] \{(\hat{u},x,u^\sharp ), (u^\sharp ,x,u^\sharp ) &{}\mid x\in I\wedge u\in F\setminus D &{}\}\,\cup \\[0pt] \{(u^\sharp ,a,\hat{v}) &{}\mid a\in V\wedge u\in F\setminus D \wedge v\in S\wedge (u,a,v)\in T &{}\} \end{array} \end{aligned}$$

The set $\hat{S}$ consists of the *original states* $\hat{u}$, the *sharp states* $u^\sharp$, and one additional state DIV. The mapping from *S* to $\hat{S}$ defined by $u\mapsto \hat{u}$ is injective and preserves acceptability and visible transitions, i.e., for any $u,v\in S$ and $a\in V$, $u{\mathop {\rightarrow }\limits ^{a}}v\;\Leftrightarrow \;\hat{u}{\mathop {\rightarrow }\limits ^{a}}\hat{v}$. It follows that paths in *B* in which all labels are visible correspond one-to-one with paths through original states in $\hat{B}$ in which all labels are visible. Note that every invisible transition in $\hat{B}$ is a self-loop or ends in a sharp state or DIV. Moreover, all transitions in $\hat{B}$ ending in a sharp state or DIV are invisible.

#### Proposition 7

For any BA *B* with alphabet *A*, and any visible set $V\subseteq A$, $\mathsf {norm}(B,V)$ is in *V*-interrupt normal form.

#### Proof

To see Definition 11(1), suppose $s_1{\mathop {\rightarrow }\limits ^{a}}s_2$. If $s_1{\mathop {\rightarrow }\limits ^{x}}s_1$, take $s_1'=s_1$. Otherwise, $s_1=\hat{u}$ for some $u\in F\setminus D$, and we can take $s_1'=u^\sharp$.

For Definition 11(2), suppose $s_1{\mathop {\rightarrow }\limits ^{x}}s_2{\mathop {\rightarrow }\limits ^{a}}s_3$. We need to show $s_1{\mathop {\rightarrow }\limits ^{a}}s_3$ and if $s_2$ is accepting then $s_1$ or $s_3$ is accepting. If $s_1=s_2$, the result is clear, so assume $s_1\ne s_2$. There are then two cases: $s_2={\textsf {DIV}}$ or $s_2=u^\sharp$ for some $u\in F\setminus D$.

If $s_2={\textsf {DIV}}$, then $a\in I$ and $s_3={\textsf {DIV}}$, and we have $s_1{\mathop {\rightarrow }\limits ^{a}}{\textsf {DIV}}$. As DIV is accepting, the desired conclusion holds.

If $s_2=u^\sharp$, then $s_1=\hat{u}$, which is accepting. There are again two cases: either $s_3=u^\sharp$ or $s_3=\hat{v}$ for some $v\in S$. If $s_3=u^\sharp$ then $a\in I$ and $\hat{u}{\mathop {\rightarrow }\limits ^{a}}u^\sharp$, as required. If $s_3=\hat{v}$, then $a\in V$ and therefore $u{\mathop {\rightarrow }\limits ^{a}}v$, hence $\hat{u}{\mathop {\rightarrow }\limits ^{a}}\hat{v}$, as required.

Definition 11(3) is clear from the definition of $\hat{T}$.    $\square$

#### Theorem 2

$\mathcal {L}(B)$ is *V*-interruptible iff $\mathcal {L}(\mathsf {norm}(B,V)) = \mathcal {L}(B)$. In particular interruptibility for Büchi Automata is decidable.

#### Proof

Let $P_1=\mathcal {L}(B)$ and $P_2=\mathcal {L}({\textsf {norm}}(B,V))$. By Proposition 7, ${\textsf {norm}}(B,V)$ is in *V*-interrupt normal form, so by Proposition 6, $P_2$ is *V*-interruptible. Hence one direction is clear: if $P_1=P_2$, then $P_1$ is *V*-interruptible.

So suppose $P_1$ is *V*-interruptible. We wish to show $P_1=P_2$. By Lemma 2, it suffices to show the two languages contain the same *V*-interrupt-free words.

Suppose $\zeta$ is a *V*-interrupt-free word in $P_1$. If $\zeta \in V^\omega$ then an accepting path $\theta$ in *B* maps to the accepting path $\hat{\theta }$ in $\hat{B}$, and $\zeta \in P_2$. So assume $\zeta \in V^*I^\omega$. Then an accepting path in *B* has a prefix $\theta$ of visible transitions ending in a state $u\in D$. That prefix corresponds to a path $\hat{\theta }$ in $\hat{B}$ ending in $\hat{u}$. As $u\in D$, $\hat{u}{\mathop {\rightarrow }\limits ^{x}}\hat{u}$ for all $x\in I$. If *u* is accepting, we get an accepting path for $\zeta$ that follows $\hat{\theta }$ and then loops at $\hat{u}$. If *u* is not accepting then $u\in D\setminus F$, and $\hat{u}{\mathop {\rightarrow }\limits ^{x}}{\textsf {DIV}}$ for all $x\in I$. Since ${\textsf {DIV}}$ is accepting and ${\textsf {DIV}}{\mathop {\rightarrow }\limits ^{x}}{\textsf {DIV}}$ for all $x\in I$, we again get an accepting path for $\zeta$ in $\hat{B}$.

Suppose now that $\zeta$ is a *V*-interrupt-free word in $P_2$. Assume $\zeta \in V^{\omega }$. An accepting path for $\zeta$ cannot pass through a sharp state or ${\textsf {DIV}}$, because only invisible transitions end in those states. So the path passes through only original states, and therefore corresponds to an accepting path in *B*.

Suppose $\zeta \in V^*I^{\omega }$. An accepting path for $\zeta$ in $\hat{B}$ consists of a prefix $\hat{\theta }$ of visible transitions followed by an infinite accepting path $\xi$ of invisible transitions. As above, $\hat{\theta }$ corresponds to a path $\theta$ in *B* ending in a state *u*.

We claim that $\xi$ cannot pass through a sharp state. This is because all invisible transitions departing from a sharp state are self loops. But sharp states are not accepting, while $\xi$ is an accepting path of invisible transitions. It follows that each transition in $\xi$ is a self-loop or terminates in DIV.

We now claim $u\in D$. For suppose the first transition in $\xi$ is a self-loop on $\hat{u}$. According to the definition of $\hat{T}$, this implies $u\in D\cup (S\setminus F)$. Hence, if $u\not \in D$ then *u* is not accepting, and all invisible transitions departing from $\hat{u}$ are self-loops, contradicting the fact that $\xi$ is an accepting path. If, on the other hand, the first transition in $\xi$ is $\hat{u}{\mathop {\rightarrow }\limits ^{x}}{\textsf {DIV}}$, for some $x\in I$, then the definition of $\hat{T}$ implies $u\in D$, establishing the claim.

So $u\in D$, i.e., there is an accepting path $\rho$ in *B* starting from *u* and consisting of all invisible transitions. The accepting path obtained by concatenating $\theta$ and $\rho$ spells a word which, projected onto *V*, equals $\zeta |_V$. Since $P_1$ is *V*-interruptible, $\zeta \in P_1$. This completes the proof that $P_1=P_2$.

The theorem reduces the problem of determining *V*-interruptibility to a problem of determining equivalence of two Büchi Automata, which can be done using language intersection, complement, and emptiness algorithms for BAs [37].    $\square$

## On-the-Fly Partial Order Reduction

### General Theory and Soundness Theorem

Let $M=(Q,A,T,q^0)$ be an LTS, $V\subseteq A$, and $B=(S,A,\delta ,S^0,F)$ a *V*-interruptible BA. The goal of on-the-fly POR is to explore a sub-automaton $R'$ of $R=M\parallel B$ with the property that $\mathcal {L}(R)=\emptyset \;\Leftrightarrow \;\mathcal {L}(R')=\emptyset$.

A function $\textsf {amp} :Q\times S\rightarrow 2^{A}$ is an *ample selector* if $\textsf {amp} (q,s)\subseteq \textsf {enabled}(M,q)$ for all $q\in Q, s\in S$. Each $\textsf {amp} (q,s)$ is an *ample set*. An ample selector determines a BA $R'={\textsf {reduced}}(R,\textsf {amp} )$ which has the same states, accepting states, and initial state as *R*, but only a subset of the transitions:

$$\begin{aligned} R'&= (Q\times S, A, \delta ', \{q^0\}\times S^0, Q\times F)\\ \delta '&= \{ ((q,s),a,(q',s')) \mid a\in \textsf {amp} (q,s) \wedge (q,a,q')\in T\wedge (s,a,s')\in \delta \}. \end{aligned}$$

We now define some constraints on an ample selector that will be used to guarantee the reduced product space has nonempty language if the full space does. First we need the usual notion of independence:

#### Definition 12

Let *M* be an LTS with alphabet *A*, and $a,b\in A$. We say *a* and *b* are *independent* if both of the following hold for all states *q* and $q'$ of *M*:

1. $$(q{\mathop {\rightarrow }\limits ^{a}}q'\wedge b\in \textsf {enabled}(M,q)) \Rightarrow b\in \textsf {enabled}(M,q')$$
2. $$q\xrightarrow {ab}q' \;\Leftrightarrow \;q\xrightarrow {ba}q'$$.

We say *a* and *b* are *dependent* if they are not independent.    $\square$

Note that, in contrast with [1], we do not assume actions are deterministic. We can now define the four constraints:

**C0**: For all $$q\in Q$$, $$s\in S$$: $$\textsf {enabled}(M,q)\ne \emptyset \Rightarrow \textsf {amp} (q,s)\ne \emptyset $$.
**C1**: For all $q\in Q$, $s\in S$: on any trace in *M* starting from *q*, no action outside of $\textsf {amp} (q,s)$ but dependent on an action in $\textsf {amp} (q,s)$ can occur without an action in $\textsf {amp} (q,s)$ occurring first.
**C2**: For all $q\in Q$, $s\in S$: if $\textsf {amp} (q,s)\ne \textsf {enabled}(M,q)$, then $\textsf {amp} (q,s)\cap V=\emptyset$.
**C3**: For all $a\in A$: on any cycle in $R'$ for which *a* is enabled in *R* at each state, there is some state (*q*, *s*) on the cycle for which $a\in \textsf {amp} (q,s)$.

#### Theorem 3

Let *M* be an LTS with alphabet *A*, $V\subseteq A$, *B* a BA with alphabet *A* in *V*-interrupt normal form, $R=M\parallel B$, and $\textsf {amp}$ an ample selector satisfying $\mathbf {C0}$–$\mathbf {C3}$. Then $\mathcal {L}(\mathsf {reduced}(R,\textsf {amp} ))=\emptyset \;\Leftrightarrow \;\mathcal {L}(R)=\emptyset$.

**Fig. 1.**
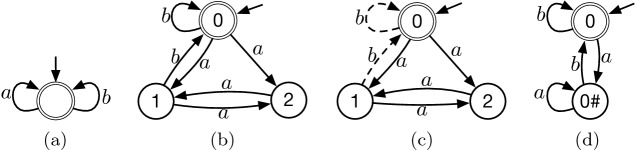
Counterexample to Theorem 3 if *B* is not in interrupt normal form: (a) the LTS *M*, (b) the BA *B* representing ${\mathbf {G}}{\mathbf {F}}b$, (c) the product space—dashed edges are in the full, but not reduced, space, and (d) the result of normalizing *B* and removing unreachable states, which also depicts the resulting full product space.

The requirement that *B* be in interrupt normal form is necessary. A counterexample when that condition is not met is given in Fig. 1. Note *a* and *b* are independent, and *a* is invisible. The ample set for product states 0 and 1 is $\{a\}$; the ample set for product state 2 is $\{a,b\}$. Hence **C3** holds because a state on the sole cycle is fully enabled. After normalizing *B* (and removing unreachable states), this problem goes away: in any reduced space, the ample sets must retain the *a*-transitions, and state $0^\sharp$ must be fully enabled since it has an *a*-self-loop, so the accepting cycle involving the two states will remain.

The remainder of this section is devoted to the proof of Theorem 3. The proof is similar to that of the analogous theorem in the state-based case [27], but some changes are necessary and we include the proof for completeness.

Let $\theta$ be an accepting path in *R*. An infinite sequence of accepting paths $\pi _0,\pi _1,\ldots$ will be constructed, where $\pi _0=\theta$. For each $i\ge 0$, $\pi _i$ will be decomposed as $\eta _i\circ \theta _i$, where $\eta _i$ is a finite path of length *i* in $R'$, $\theta _i$ is an infinite path, and $\eta _i$ is a prefix of $\eta _{i+1}$. For $i=0$, $\eta _0$ is empty and $\theta _0=\theta$.

Assume $i\ge 0$ and we have defined $\eta _j$ and $\theta _j$ for $j\le i$. Write

1 $$\begin{aligned} \theta _i \,\, = \,\, \langle q_0,s_0\rangle \xrightarrow {a_1} \langle q_1,s_1\rangle \xrightarrow {a_2} \cdots \end{aligned}$$

Then $\eta _{i+1}$ and $\theta _{i+1}$ are defined as follows. Let $E=\textsf {amp} (q_0,s_0)$. There are two cases:

*Case 1:* $a_1\in E$. Let $\eta _{i+1}$ be the path obtained by appending the first transition of $\theta _i$ to $\eta _i$, and $\theta _{i+1}$ the path obtained by removing the first transition from $\theta _i$.

*Case 2:* $a_1\not \in E$. Then there are two sub-cases:

*Case 2a:* Some operation in *E* occurs in $\theta _i$. Let *n* be the index of the first such occurrence. By **C1**, $a_j$ and $a_n$ are independent for $1\le j<n$. By repeated application of the independence property, there is a path in *M* of the form

$$ q_0 {\mathop {\rightarrow }\limits ^{a_n}} q_1' {\mathop {\rightarrow }\limits ^{a_1}} q_2' {\mathop {\rightarrow }\limits ^{a_2}} \cdots {\mathop {\rightarrow }\limits ^{a_{n-2}}} q_{n-1}' {\mathop {\rightarrow }\limits ^{a_{n-1}}} q_n {\mathop {\rightarrow }\limits ^{a_{n+1}}} q_{n+1} {\mathop {\rightarrow }\limits ^{a_{n+2}}} \cdots . $$

By **C2**, $a_n$ is invisible. By Definition 11, *B* has an accepting path of the form

$$ s_0 {\mathop {\rightarrow }\limits ^{a_n}} s_0' {\mathop {\rightarrow }\limits ^{a_1}} s_1 {\mathop {\rightarrow }\limits ^{a_2}} \cdots {\mathop {\rightarrow }\limits ^{a_{n-2}}} s_{n-2} {\mathop {\rightarrow }\limits ^{a_{n-1}}} s_{n-1} {\mathop {\rightarrow }\limits ^{a_{n+1}}} s_{n+1} {\mathop {\rightarrow }\limits ^{a_{n+2}}} \cdots . $$

Composing these two paths yields a path in *R*. Removing the first transition (labeled $a_n$) yields $\theta _{i+1}$. Appending that transition to $\eta _i$ yields $\eta _{i+1}$.

*Case 2b:* No operation in *E* occurs in $\theta _i$. By **C0**, *E* is nonempty. Let $b\in E$. By $\mathbf{C2 }$, every action in $\theta _i$ is independent of *b*. As in the case above, we obtain a path in *R*

$$ \langle q_0,s_0\rangle {\mathop {\rightarrow }\limits ^{b}} \langle q_1',s_0'\rangle {\mathop {\rightarrow }\limits ^{a_1}} \langle q_2',s_1\rangle {\mathop {\rightarrow }\limits ^{a_2}} \langle q_3',s_2\rangle {\mathop {\rightarrow }\limits ^{a_3}} \cdots . $$

and define $\theta _{i+1}$ and $\eta _{i+1}$ as above.

Let $\eta$ be the limit of the $\eta _i$, i.e., $\eta (i)=\eta _{i+1}(i)$. It is clear that $\eta$ is an infinite path in $R'$, but we must show it passes through an accepting state infinitely often. To see this, define integers $d_i$ for $i\ge 0$ as follows. Let $\xi _i=s_0s_1\cdots$ be the sequence of BA states traced by $\theta _i$. Let $d_i$ be the minimum $j\ge 0$ such that $s_j$ is accepting. Note that $d_i=0$ iff ${\mathsf {last}}(\eta _i)$ is accepting.

Suppose $i\ge 0$ and $d_i>0$. If Case 1 holds, then $d_{i+1}=d_i-1$, since $\xi _{i+1}=\xi _i^1$. It is not hard to see that if Case 2 holds, $d_{i+1}\le d_i$. Note that in Case 2a, if $d_i=n$, the accepting state $s_n$ is removed, but Definition 11(2) guarantees that at least one of $s_{n-1}$ and $s_{n+1}$ is accepting. In the worst case ($s_{n-1}$ is not accepting), we still have $d_{i+1}=n$.

We claim there are an infinite number of $i\ge 0$ such that Case 1 holds. Otherwise, there is some $i>0$ such that Case 2 holds for all $j\ge i$. Let *a* be the first action in $\theta _i$. Then for all $j\ge i$, *a* is the first action of $\theta _j$ and *a* is not in the ample set of ${\mathsf {last}}(\eta _j)$. Since the number of states of *R* is finite, there is some $k>i$ such that ${\mathsf {last}}(\eta _k)={\mathsf {last}}(\eta _i)$. Hence there is a cycle in $R'$ for which *a* is always enabled but never in the ample set, contradicting **C3**.

If $\eta$ does not pass through an accepting state infinitely often, there is some $i\ge 0$ such that for all $j\ge i$, ${\mathsf {first}}(\theta _j)$ is not accepting. But then $(d_j)_{j\ge i}$ is a nondecreasing sequence of positive integers which strictly decreases infinitely often, a contradiction.

### Ample Sets for a Parallel Composition of LTSs

We now describe the specific method used by McRERS to select ample sets. Since this method is similar to existing approaches, such as [32, Algorithm 4.3], we just outline the main ideas.

Let $n\ge 1$, $P=\{1,\ldots ,n\}$, and let $M_1,\ldots , M_n$ be LTSs over ${\textsf {Act}}$. Write $M_i=(Q_i,A_i,\rightarrow _i,q^0_i)$ and

$$ M = M_1 \parallel \cdots \parallel M_n = (Q, A, \rightarrow , q^0). $$

For $a\in A$, let $\textsf {procs} (a)=\{i\in P\mid a\in A_i\}$. It can be shown that if *a* and *b* are dependent actions, then $\textsf {procs} (a)\cap \textsf {procs} (b)\ne \emptyset$.

Let $q=(q_1,\ldots ,q_n)\in Q$ and $E_i=\textsf {enabled}(M_i,q_i)$ for $i\in P$. Let

$$ R_q=\{(i,j)\in P\times P\mid E_i\cap A_j\ne \emptyset \}. $$

Suppose $C\subseteq P$ is closed under $R_q$, i.e., for all $i\in C$ and $j\in P$, $(i,j)\in R_q \Rightarrow j\in C$. This implies that if $a\in E_i$ for some $i\in C$ then $\textsf {procs} (a)\subseteq C$. Define

$$ \textsf {enabled}(C,q) = \textsf {enabled}(M,q)\cap \bigcup _{i\in C} A_i. $$

Let $E=\textsf {enabled}(C,q)$. Note $E\subseteq \bigcup _{i\in C}E_i$. Hence for any $a\in E$, $\textsf {procs} (a)\subseteq C$.

#### Lemma 3

On any trace in *M* starting from *q*, no action outside of *E* but dependent on an action in *E* can occur without an action in *E* occurring first.

#### Proof

Let $\zeta$ be a trace in *M* starting from *q*, such that no element of *E* occurs in $\zeta$. We claim no action involving *C* (i.e., an action *a* for which $\textsf {procs} (a)\cap C\ne \emptyset$) can occur in $\zeta$. Otherwise, let *x* be the first such action. Then $x\in E_i$, for some $i\in C$, so $\textsf {procs} (x)\subseteq C$. As $x\not \in E$, $x\not \in \textsf {enabled}(M,q)$. So some earlier action *y* in $\zeta$ caused *x* to become enabled, and therefore $\textsf {procs} (x)\cap \textsf {procs} (y)\ne \emptyset$, hence $\textsf {procs} (y)\cap C\ne \emptyset$, contradicting the assumption that *x* was the first action involving *C* in $\zeta$.

Now any action *b* dependent on an action $a\in E$ must satisfy $\textsf {procs} (a)\cap \textsf {procs} (b)$ is nonempty. Since $\textsf {procs} (a)\subseteq C$, $\textsf {procs} (b)\cap C$ is nonempty. Hence no action dependent on an action in *E* can occur in $\zeta$.    $\square$

We now describe how to find an ample set in the context of NDFS. Let (*q*, *s*) be a new product state that has just been pushed onto the outer DFS stack. The relation $R_q$ defined above gives *P* the structure of a directed graph. Suppose that graph has a strongly connected component $C_0$ such that all of the following hold for $E=\textsf {enabled}(C_0,q)$:

1. $$E\ne \emptyset $$,
2. $$E\cap V=\emptyset $$,
3. $\textsf {enabled}(C',q)=\emptyset$ for all SCCs $C'$ reachable from $C_0$ other than $C_0$, and
4. *E* does not contain a “back edge”, i.e., if $(q,s){\mathop {\rightarrow }\limits ^{a}}\sigma$ for some $a\in E$ and $\sigma \in Q\times S$, then $\sigma$ is not on the outer DFS stack.

Then set $\textsf {amp} (q,s)=E$. If no such SCC exists, set $\textsf {amp} (q,s)=\textsf {enabled}(M,q)$. It follows that **C0**–**C4** hold. Note that the union *C* of all SCCs reachable from $C_0$ is closed under $R_q$, and $\textsf {enabled}(C,q)=E$, so Lemma 3 guarantees **C1**. For **C3**, we actually have the stronger condition that in any cycle in the reduced space, at least one state is fully enabled. In our implementation, the SCCs are computed using Tarjan’s algorithm. Among all SCCs $C_0$ satisfying the conditions above, we choose one for which $|\textsf {enabled}(C_0,q)|$ is minimal.

One known issue when combining NDFS with on-the-fly POR is that the inner DFS must explore the same subspace as the outer DFS, i.e., $\textsf {amp}$ must be a deterministic function of its input (*q*, *s*) [18]. To accomplish this, McRERS stores one additional integer *j* in the state: *j* is the root node of the SCC $C_0$, or $-1$ if the state is fully enabled. The outer search saves *j* in the state, and the inner search uses *j* to reconstruct the SCC $C_0$ and the ample set *E*.

## Related Work

There has been significant earlier research on the use of partial order reduction to model check LTSs (or the closely related concept of process algebras); see, e.g., [14, 16, 30–33, 35]. To understand how this previous work relates to this paper, we must explain a subtle, but important, distinction concerning how a property is specified. In much of this literature, a property of an LTS with alphabet *A* is essentially a pair $\pi =(V, T)$, where $V\subseteq A$ is a set of visible actions and *T* is a set of (finite and infinite) words over *V*. A property in this sense specifies acceptable behaviors *after invisible actions have been removed*. (See, e.g., Def. 2.4 and preceding comments in [32].) We can translate $\pi$ to a property *P* in our sense by taking its inverse image under the projection map:

$$ P=\{\zeta \in A^\omega \mid \zeta |_V\in T\}. $$

Note that *P* *is* *V-interruptible by definition*. Hence the need to distinguish interruptible properties does not arise in this context.

Much of the earlier work on POR for LTSs deals with the “offline” case, i.e., the construction of a subspace of *M* that preserves certain classes of properties. In contrast, Theorem 3 deals with an on-the-fly algorithm, i.e., the construction of a subspace of $M\parallel B$. The on-the-fly approach is an essential optimization in model checking, but recent work in the state-based formalism has shown that offline POR schemes do not always generalize easily to on-the-fly algorithms [27].

One work that does describe an on-the-fly model checking algorithm for LTSs is [32] (see also [17], which deals with the same ideas in a state formalism). The property is specified by a *tester process* *B*. Consistent with the notion of *property* described above, the alphabet of *B* does not include the invisible actions. Hence, in the parallel composition $M\parallel B$, the tester does not move when *M* executes an invisible action. In order to specify both finite and infinite words of visible actions, the tester has two kinds of accepting states: “livelock monitor states” and “infinite trace monitor states.” (Two additional classes of states for detecting other kinds of violations are not relevant to the discussion here.) A version of the stubborn set theory is used to define the reduced space, and a special condition is used to solve the “ignoring problem” (instead of our **C3**). It would be interesting to compare this algorithm with the one described here.

There are many algorithms for reducing or even minimizing the size of an LTS while preserving various properties, e.g., *bisimulation equivalence* [8] or *divergence preserving bisimilarity* [6]. These algorithms could be applied to the individual components of a parallel composition (taking all visible and communication actions to be “visible”), as a preprocessing step before beginning the model checking search. An exploration of these algorithms, and how they impact POR, is beyond the scope of this paper, but we hope to explore that avenue in future work.

The RERS Challenge [9, 19–21] is an annual event involving a number of different categories of large model checking problems. The “parallel LTL category,” offered from 2016 on, is directly relevant to this paper. Each problem in that category consists of a Graphviz “dot” file specifying an LTS as a parallel composition, and a text file containing 20 LTL formulas. The goal is to identify the formulas satisfied by the LTS. The solutions are initially known only to the organizers, and are published after the event. The RERS semantics for LTSs, LTL, and satisfiability are exactly the same as in this paper.

The methods for generating the LTS and the properties are complicated, and have varied over the years, but are designed to satisfy certain hardness guarantees. The approach described in [29] is “...based on the weak refinement ...of convergent systems which preserves an interesting class of temporal properties.” It can be seen that the properties preserved by weak refinement are exactly the interruptible properties. While [29] does not describe a method for determining whether a property is interruptible, the authors have informed us that they developed a sufficient condition for an LTL formula to be interruptible, and used this in combination with a random method to generate the formulas for 2016 and 2019. Our analysis (Sect. 6) confirms that all formulas from 2016 and 2019 are interruptible, while 2017 and 2018 contain some non-interruptible formulas.

There is a well-known way to translate a system and property expressed in an action-based formalism to a state-based formalism. The idea is to add a shared variable $\textit{last}$ which records the last action executed. An LTL formula over actions can be transformed to one over states by replacing each action *a* with the predicate $\textit{last}=a$. This is the approach taken in the Promela representations of the parallel problems provided with the RERS challenges.

This translation is semantics-preserving but performance-destroying. Every transition writes to the shared variable $\textit{last}$, so any state-based POR scheme will assume that no two transitions commute. Furthermore, since the property references $\textit{last}$, all transitions are visible. This effectively disables POR, even when the property is stutter-invariant, as can be seen in the poor performance of Spin on the RERS Promela models (Sect. 6). It is possible that there are more effective Spin translations; [34, §2.2], for example, suggests not updating $\textit{last}$ on invisible actions, and adding a global boolean variable that is flipped on every visible action (in addition to updating $\textit{last}$). We note that this would also require modifying the LTL formula, or specifying the property in some other way. In any case, it suggests another interesting avenue for future work.

## Experimental Results and Conclusions

We implemented a model checker named McRERS based on the algorithms described in this paper. McRERS is a library and set of command line tools. It is written in sequential C and uses the Spot library [4] for several tasks: (1) determining equivalence of LTL formulas, (2) determining language equivalence of BAs, and (3) converting an LTL formula to a BA. The source code for McRERS as well as all artifacts related to the experiments discussed in this section, are available at https://vsl.cis.udel.edu/cav2020. The experiments were run on an 8-core 3.7GHz Intel Xeon W-2145 Linux machine with 256 GB RAM, though McRERS is a sequential program and most experiments required much less memory.

As described in Sect. 5, each edition of RERS includes a number of problems, each of which comes with 20 LTL formulas. The numbers of problems for years 2016–2019 are, in order, 20, 15, 3, and 9, for a total of 47 problems, or $47*20=940$ distinct model checking tasks. (Some formulas become identical after renaming propositions.) We used the McRERS *property analyzer* to analyze these formulas to determine which are interruptible; the algorithm used is based on Theorem 1. The results show that all formulas from 2016 and 2019 are interruptible, which agrees with the expectations of the RERS organizers. In 2017, 22 of the 300 formulas are not interruptible; these include

,
, and
.

In 2018, 3 of the 60 formulas are not interruptible. In summary, only 25 of the 940 tasks involve non-interruptible formulas. The total runtime for the analysis of all 940 formulas was 6 s.

We next used the McRERS *automaton analyzer* to create BAs from each of the interruptible formulas, and then to determine which of these Spot-generated BAs was not in interrupt normal form. This uses a straightforward algorithm that iterates over all states and checks the conditions of Definition 11. For each BA not in normal form, the analyzer transforms it to normal form using function ${\textsf {norm}}$ of Sect. 3.4. Interestingly, all of the Spot-generated BAs in 2016 and 2019 were already in normal form. Four of the BAs from interruptible formulas in 2017 were not in normal form; all of these formulas had the form ${\mathbf {F}}[a\vee ((\lnot b){\mathbf {W}}c)]$. In 2018, 6 interruptible formulas have non-normal BAs; these formulas have several different non-isomorphic forms, some of which are quite complex. The details can be seen on the online archive. The total runtime for this analysis (including writing all BAs to a file) was 11 s.

The McRERS model checker parses RERS “dot” and property files to construct an internal representation of a parallel composition $M=M_1\parallel \cdots \parallel M_n$ of LTSs and a list of LTL formulas. Each formula *f* is converted to a BA *B*; if *f* is interruptible and *B* is not already in normal form, *B* is transformed to normal form. The NDFS algorithm is used to determine language emptiness, and if *f* is interruptible, the POR scheme described in Sect. 4 is also used. States are saved in a hash table.

One other simple optimization is used regardless of whether *f* is interruptible. Let $\alpha M$ denote the set of actions labeling at least one transition in *M*, and define $\alpha B$ similarly. If $\alpha M\ne \alpha B$, then all transitions labeled by an action in $(\alpha M\setminus \alpha B)\cup (\alpha B\setminus \alpha M)$ are removed from the $M_i$ and *B*; all unreachable states and transitions in the $M_i$ and *B* are also removed. This is repeated until $\alpha M=\alpha B$.

**Fig. 2.**
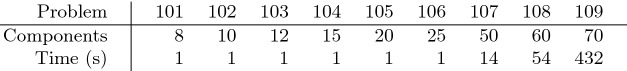
Time to solve RERS 2019 parallel LTL problems using McRERS. Each problem comprises 20 LTL formulas. Memory limited to 8 GB. Rows: problem number, number of components in the LTS, and total McRERS wall time rounded up to nearest second.

We applied the model checker to all problems in the 2019 benchmarks. Interestingly, all 180 tasks completed, with the correct results, using at most 8 GB RAM; the times are given in Fig. 2.

**Fig. 3.**
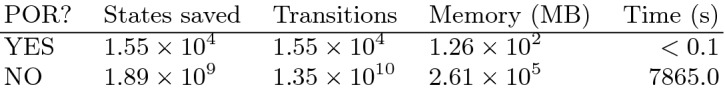
Performance impact of POR on solving RERS 2019 problem 106, formula 1, $(\texttt {\small a6}\rightarrow {\mathbf {F}}\texttt {\small a7}){\mathbf {W}}(\texttt {\small a7}\vee \texttt {\small a88})$.

We also ran these problems with POR turned off, to measure the impact of that optimization. As is often the case with POR schemes, the difference is dramatic. The non-POR tests ran out of memory on our 256 GB machine after problem 106. We show the resources consumed for a representative task in Fig. 3; this property holds, so a complete search is required. In terms of number of states or time, the performance differs by about 5 orders of magnitude.

**Fig. 4.**
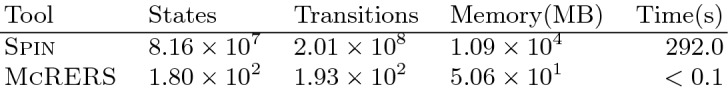
Performance of Spin v6.5.1 and McRERS on RERS 2019 problem 101, property 1. Both tools used POR. Spin used -DCOLLAPSE for state compression and -m100000000 for search depth bound.

As explained in Sect. 5, the RERS Spin models can not be expected to perform well. We ran the latest version of Spin on these using -DCOLLAPSE compression. We show the result for just the first task in Fig. 4. There is at least a 4 order of magnitude performance difference (measured in states or time) between the tools. An examination of Spin’s output in verbose mode reveals the problem to be as described in Sect. 5: the full set of enabled transitions is explored at each transition due to the update of the shared variable.

The 2016 RERS problems are more challenging for McRERS. The problems are numbered from 101 to 120. To scale beyond problem 111, with a memory bound of 256 GB, additional reduction techniques, such as the component minimization methods discussed in Sect. 5, must be used. We plan to carry out a thorough study of those methods and how they interact with POR.
